# Splenic artery pseudoaneurysm rupture post-laparoscopic sleeve gastrectomy

**DOI:** 10.1093/jscr/rjae752

**Published:** 2024-11-25

**Authors:** Stephanie M Babic, Roshan N Ramachandran

**Affiliations:** Department of General Surgery, Albany Health Campus, Western Australia Country Health Service, 30 Warden Ave, Spencer Park, Western Australia, 6330, Australia; Department of General Surgery, Albany Health Campus, Western Australia Country Health Service, 30 Warden Ave, Spencer Park, Western Australia, 6330, Australia

**Keywords:** splenic artery pseudoaneurysm, bariatric surgery, laparoscopic sleeve gastrectomy, rural surgery

## Abstract

A splenic artery pseudoaneurysm (SAP) is a rare vascular entity that is becoming increasingly recognized as a potential complication of bariatric surgery. This is a case of a 36-year-old woman brought by ambulance to a regional emergency department with abdominal pain, collapse, and gross haemodynamic instability 2 years post-laparoscopic sleeve gastrectomy. She received aggressive resuscitation in the emergency department but could not be stabilized and so underwent an emergency laparotomy. Intra-operatively, she was found to have a ruptured SAP with active bleeding, which was managed with a splenectomy. A high index of suspicion is required in any patient presenting with abdominal pain and circulatory collapse in the context of previous bariatric surgery. In rural or regional settings without immediate access to interventional radiology services, the most appropriate management option will almost invariably be an emergency laparotomy and splenectomy due to the risk of deterioration during patient transfer.

## Introduction

A splenic artery pseudoaneurysm (SAP) is a rare vascular entity with few reported cases in the literature. However, SAPs are becoming a recognized, albeit uncommon, complication post-laparoscopic sleeve gastrectomy (LSG) [1, 2]. In 2023, the Medicare Benefits Schedule recorded 21 044 bariatric procedures performed in Australia [3]. LSGs accounted for 80% of primary bariatric surgical procedures in the Medicare database [3]. Therefore, despite its rarity, an SAP is a complication one must consider when a patient presents with acute abdominal pain in the context of a previous LSG. This is particularly pertinent if the abdominal pain fits with the clinical picture of the ‘double rupture’ phenomenon, with initial abdominal pain caused by SAP rupture into the lesser sac, subsequent tamponade, and then an increase in abdominal pain and development of haemorrhagic shock as the haematoma decompresses into the peritoneal cavity [4]. This is a case of a haemodynamically unstable patient with a ruptured SAP who required an emergency laparotomy and splenectomy on a background of an LSG 2 years prior.

## Case

**Figure 1:**
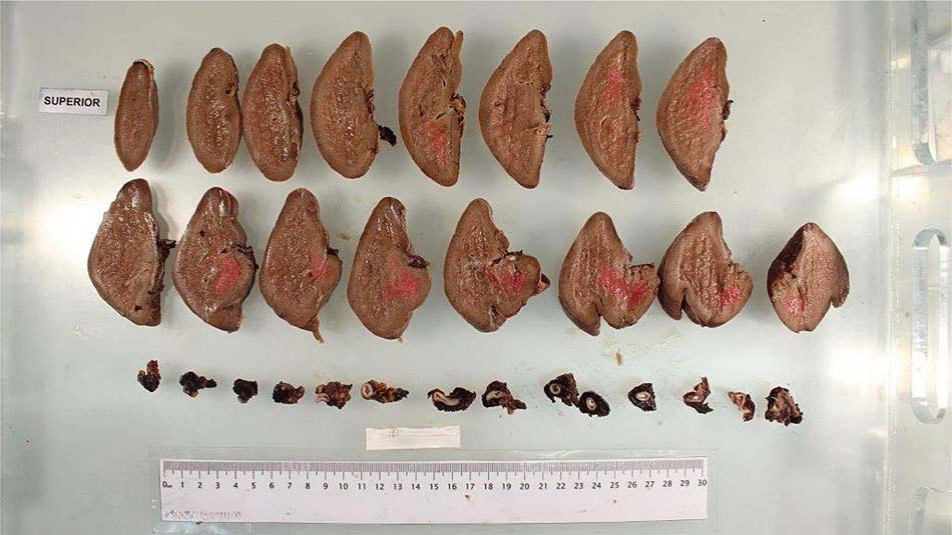
Patient’s spleen and ruptured splenic artery pseudoaneurysm.

A 36-year-old woman was brought by ambulance to a regional hospital after experiencing mild abdominal pain that morning, followed by a sudden acute increase in abdominal pain and collapse a few hours later. She had a background of a LSG 2 years prior and no other significant comorbidities. On arrival, she was pale and minimally responsive. She had a thready pulse and was tachycardic at 120 bpm and hypotensive at 90/40 mmHg. A bedside ultrasound in the emergency department demonstrated significant free fluid in the abdomen. Her venous blood gas showed pH 7.27, pCO2 52 mmHg, bicarbonate 24 mmol/L, and lactate 3.4 mmol/L. Her full blood count showed haemoglobin 80 g/L. The hospital’s massive transfusion protocol was initiated. She was intubated in the department and a central venous catheter and radial arterial line were placed. She was taken for an emergency laparotomy and splenectomy without imaging due to her haemodynamic instability. Intra-operatively, there was active bleeding from a ruptured SAP and approximately 3 litres of intra-abdominal blood. There was a large haematoma in the lesser sac which extended into the retroperitoneum but was not expanding. The tail of pancreas was partially transected during control of haemostasis, as the pancreas was lifted by the haematoma in the lesser sac. An AbThera vacuum-assisted closure dressing was applied. She was then transferred via the Royal Flying Doctor Service to an intensive care unit within a tertiary hospital centre. She underwent a relook laparotomy, washout, and definitive closure the next day. Her post-operative recovery was complicated by pancreatitis, ileus, a reactive thrombocytosis, and a transient acute kidney injury, all of which resolved by discharge. Post-splenectomy vaccines were administered, and she was discharged on life-long prophylactic low-dose amoxicillin 16 days after her initial presentation. Figure 1 was obtained from the pathology team and demonstrates slices of spleen and the ruptured SAP.

## Discussion

Development of an SAP is a rare but recognized potential complication post-LSG. Recently, Mousavimaleki *et al*. [1] conducted a systematic review analyzing splenic complications post-bariatric surgery. The authors noted that the incidence of rare complications is increasing due to the rise in bariatric surgery worldwide. They found that the main splenic complications were abscess, infarct, SAP, and splenic rupture. 11% of 53 patients studied developed an SAP post-bariatric surgery.

The scarcity of reported SAPs in the literature renders it difficult to detail a ‘typical’ presentation of SAP rupture. In contrast with splenic artery aneurysms (SAAs), which are usually asymptomatic, an SAP almost always presents with symptoms, with only 2.5% of SAPs being identified incidentally [4]. In this case, the primary presenting symptom was abdominal pain with the double rupture phenomenon. However, a ruptured SAP can present in a variety of ways, including with gastrointestinal bleeding if it ruptures into the colon, or haemosucus pancreaticus if it ruptures into the pancreatic duct [5]. Another factor to consider regarding presentation is the time of SAP occurrence post-LSG. Time from index operation to presentation of an SAP has been theorized to range from 9 to 118 days [6], but time to symptoms or rupture may occur several years post-LSG and can be highly variable [6, 7].

The aetiology of SAP formation is also unclear. Several aetiologies have been posited in previous papers, including gastric fistulae, constant irritation from the staple line, and iatrogenic injury during surgery [6–9]. However, the risk of rupture is clear, with SAPs having a 37% chance of rupture, regardless of size, and a 90% mortality rate if left untreated [4]. This is compared with a 2%–3% risk of rupture for SAAs [4]. The cause of SAP formation in this case was unclear from the clinical findings and histopathology report.

The significant rupture risk and high mortality rate of SAPs mandate that all are treated, even if asymptomatic. The choice of treatment varies according to the haemodynamic status of the patient. Pre-treatment computed tomography imaging followed by angioembolization under an interventional radiologist is generally the first-line choice for a haemodynamically stable patient with ready access to the resources required [1]. However, angioembolization has a higher failure rate for treatment of SAPs than splenectomy, regardless of the diameter of the aneurysm [10]. Further, many regional settings do not have easy access to interventional radiology. In this case, the haemodynamic instability of the patient, coupled with being in a regional centre, required emergency operative intervention.

## Conclusion

SAPs are rare vascular entities that pose challenges in both diagnosis and management. The incidence of SAPs is increasing due to the rise in bariatric surgery. A high index of suspicion is therefore required in patients presenting with abdominal pain or gastrointestinal bleeding in the context of previous bariatric surgery, particularly if the patient is haemodynamically unstable. Management options for a ruptured SAP will be dictated by the patient’s haemodynamic status and access to interventional radiology and operative resources. In rural and regional settings, the most appropriate management option is an emergency laparotomy and splenectomy, due to the risk of clinical deterioration if the patient is transferred to another centre for angioembolization, even if the patient is stable pre-transfer.
